# Real-Time Control of a Focus Tunable Lens for Presbyopia Correction Using Ciliary Muscle Biopotentials and Artificial Neural Networks

**DOI:** 10.3390/bioengineering12111228

**Published:** 2025-11-10

**Authors:** Bishesh Sigdel, Sven Schumayer, Sebastian Kaltenstadler, Eberhart Zrenner, Volker Bucher, Albrecht Rothermel, Torsten Straßer

**Affiliations:** 1 Institute for Ophthalmic Research, University of Tuebingen, 72076 Tuebingen, Germany; bishesh.sigdel@uni-tuebingen.de (B.S.); ez@uni-tuebingen.de (E.Z.); 2 Faculty Mechanical and Medical Engineering (MME), Institute for Microsystems Technology (iMST), Furtwangen University, 78120 Furtwangen, Germany; volker.bucher@hs-furtwangen.de; 3 Institute of Microelectronics, University of Ulm, 89081 Ulm, Germany; sebastian.kaltenstadler@uni-ulm.de (S.K.); albrecht.rothermel@uni-ulm.de (A.R.); 4 University Eye Hospital Tuebingen, 72076 Tuebingen, Germany

**Keywords:** accommodation, biopotential signals, deep neural network, dynamic control, machine learning, multi-layer perceptron, presbyopia, tunable lens

## Abstract

Ageing results in the progressive loss of near vision, known as presbyopia, which impacts individuals and society. Existing corrective methods offer only partial compensation and do not restore dynamic focusing at varying distances. This work presents a closed-loop correction system for presbyopia, employing biopotential signals from the ciliary muscle and an artificial neural network to predict the eye’s accommodative state in real time. Non-invasive contact lens electrodes collect biopotential data, which are preprocessed and classified using a multi-layer perceptron. The classifier output guides a control system that adjusts an external focus-tunable lens, enabling both accommodation and disaccommodation similar to a young eye. The system demonstrated an accuracy of 0.79, with F1-scores of 0.78 for prediction of accommodation and 0.77 for disaccommodation. Using the system in two presbyopic subjects, near visual acuity improved from 0.28 and 0.38 to 0.04 and −0.03 logMAR, while distance acuity remained stable. Despite challenges such as signal quality and individual variability, the findings demonstrate the feasibility of restoring near-natural accommodation in presbyopia using neuromuscular signals and adaptive lens control. Future research will focus on system validation, expanding the dataset, and pre-clinical testing in implantable devices.

## 1. Introduction

Accommodation refers to the eye’s capability to focus at various depths, enabling clear vision of both near and distant objects. Presbyopia, a condition characterized by the gradual age-related decline in accommodative ability due to the stiffening of the crystalline lens of the eye [1,2,3], prevents individuals from seeing near objects clearly. It is estimated to have a significant global impact, affecting approximately 1.8 billion people as of 2015, and its prevalence is expected to rise further with increasing human life expectancy [4].

While the mechanism underlying accommodation and presbyopia has been extensively studied for over a century, it remains a subject of ongoing investigation and debate [1]. Duane showed in 1912 that the amplitude of accommodation decreases steadily from later childhood, with little impairment in the accommodative ability until around age 40 at which point the subjective amplitude of accommodation falls below the level required for normal near vision [5]. The progression of presbyopia generally exhibits little individual variation, such that by around 55 years of age, focusing on objects within arm’s reach becomes highly challenging, and by 70 years old, virtually no accommodation is possible [1,5].

The accommodation process is driven by the contraction and expansion of the ring-shaped ciliary muscle, that is connected to the crystalline lens through the zonular fibers. Most of the accepted models of accommodation are based on the classical theory proposed by Helmholtz in 1855 [6,7,8]. According to Helmholtz’s theory and subsequent research, when the ciliary muscle contracts, it moves anteriorly and radially inward, thereby reducing the tension on the zonular fibers [9]. This allows the elastic lens to become more spherical, increasing its optical power. Conversely, during disaccommodation, relaxation of the ciliary muscle leads to posterior and radial outward movement. This action increases tension on the zonular fibers, causing the lens to flatten. These dynamic changes in lens shape, driven by the contraction and relaxation of the ciliary muscle, facilitate the focusing of light from various distances onto the retina, enabling clear vision at different viewing distances in a young eye.

Theories on the onset of presbyopia have evolved over time, with explanations focusing on changes in both the crystalline lens and other components involved in accommodation [10]. However, the emphasis has shifted to alterations within the lens itself, such as increased stiffness, size, and decreased elasticity, which limit the lens’s ability to change shape for accommodation [11]. Although the primary cause of presbyopia remains debated, it is widely accepted that the ciliary muscle function is at least partially preserved in presbyopes [12,13,14]. In a study by Tabernero et al. [15], high-speed measurements of lens movement in pseudophakic subjects revealed that lens wobbling increased when fixating on near distances, providing strong evidence of ciliary muscle contraction even in late-stage presbyopia.

Although various optical methods, surgical and pharmacological interventions exist to correct presbyopia, none can fully restore the eye’s natural accommodation capabilities [14,16,17]. Spectacles and contact lenses are common non-invasive options that can provide focused vision at near distances. Still, they do not address the underlying cause of presbyopia and may require frequent adjustments as the condition progresses [14,16]. Surgical approaches, such as refractive lens exchange or corneal refractive procedures, offer more permanent solutions but carry inherent risks and do not replicate the dynamic range of accommodation seen in younger eyes [14,16]. Pharmacological interventions have garnered increasing attention in recent years, with Pilocarpine hydrochloride 1.25% (Vuity; AbbVie Inc., North Chicago, IL, USA) being approved by the Food and Drug Administration (FDA) for presbyopia in the United States, and other clinical trials underway [18,19].

The demand for actively controllable lenses to correct presbyopia has been acknowledged, leading to the development of several approaches. Karkhanis et al. [20] proposed an accommodating lens that adjusts its power based on distance sensor input. However, reliance on distance sensing alone may not accurately reflect the eye’s accommodative requirements. Agarwala et al. [21] developed more sophisticated accommodating spectacles that employed a Light Detection and Ranging (LiDAR) sensor and gaze tracking to better assess accommodative demand. Despite the technically interesting approaches, the practical adoption of these technologies in daily life seems challenging due to the bulky design, high energy consumption and potential inaccuracies in estimated accommodation demand from distance sensor data. Alternative architectures, such as matrices of tunable liquid-crystal lenses (MTLCLs), could enable dynamic correction with reduced size and power suitable for contact or intraocular lens (IOL) implementation [22]. Even though a plethora of ideas for treatment of presbyopia were patented, to the best of our knowledge, no solution for true restoration of accommodation has been developed for presbyopia.

For binocular near vision, three aspects of accommodation are triggered, the constriction of pupils, change in lens power through ciliary muscle contraction and binocular convergence, also known as the ‘near triad’ of accommodation [1,23]. Control models for accommodation involving closed-loop negative-feedback have been posited by Toates in the 1970s [24,25] and widely accepted in modern literature [11]. These models highlight the feedback mechanisms in the autonomic nervous system crucial for maintaining dynamic clear vision, an aspect that current corrections do not offer [11,18]. Although mechanically accommodating intraocular lenses have been explored as a potential solution to restore accommodation by capturing ciliary muscle movements and offering different optical power through their change in shape or position within the eye, these designs have encountered substantial difficulties in effectively converting ciliary muscle activity into corresponding refractive power adjustments [14,18]. Further IOL designs that enable electronic control of an artificial lens using real-time signals from the near triad are reportedly under development [26].

Investigations into the electrophysiological properties of the ciliary muscle were initiated decades ago, yet the topic has received limited attention in current research [27,28,29,30]. Utilizing the electrical signals of the ciliary muscle has the potential to offer a more physiological but simpler approach to presbyopia correction by leveraging the eye’s natural control pathways. In a prior study [31], we conducted non-invasive measurements of ciliary muscle biopotential signals during accommodation in emmetropic subjects, utilizing concentric differential electrodes integrated into a scleral contact lens [32]. The results demonstrated a significant correlation between accommodation demand and the measured biopotential signals, enabling the estimation of the eye’s accommodative demand and real-time control of an artificial lens. We have developed a predictive system, validated through data from a retrospective analysis [31], that determines the refractive state of a tunable lens using signal processing, machine learning and decision support algorithm. The system is designed for low latency, facilitating dynamic adjustments to higher optical powers when the wearer attempts to focus on near objects and vice versa, closely mimicking the natural accommodation process. Additionally, the system has been tested in a preliminary study with two presbyopic volunteers.

## 2. Materials and Methods

### 2.1. Measurement and Recording System

The sensing system utilized a scleral contact lens incorporating two concentric gold rings, contacting the eye surface just above the ciliary muscle. These gold rings served as differential electrodes, with the inner ring being placed over the limbus, where the apex of the ciliary muscle lies underneath the sclera. Ciliary muscle contraction for accommodation results in the increase in thickness in the apex, and most of the change in the muscle geometry occurs within 1.5 mm distance from the scleral spur [33]. The active inner ring electrode position is placed close to the apex while the reference outer ring is placed farther away on the sclera to measure biopotential changes associated with muscle contraction during accommodation [31]. Schumayer et al. (2022) [32] detail the manufacturing process and rationale for employing scleral lenses, which facilitate an increased distance and more peripheral placement of the reference electrode, thereby enabling the recording of biopotentials with higher amplitude. An implantable version of the measurement system and software, developed specifically for capturing ciliary muscle potentials intraocularly in a preclinical model, is described in Kaltenstadler et al. [34]. However, for the human subject study using noninvasive contact lens electrodes [35], a larger, non-implantable printed circuit board (PCB) was employed.

### 2.2. Data Selection and Annotation for Supervised Machine Learning

**Figure 1:**
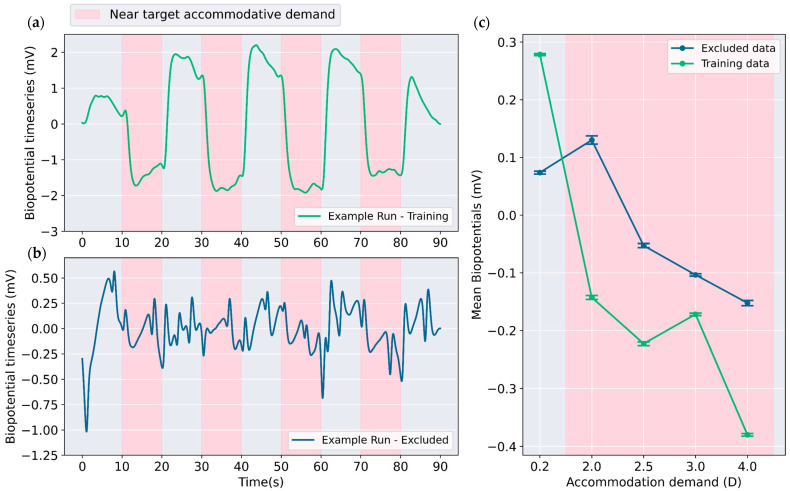
(**a**) Example of an optimal measurement run selected for training. The region shaded in pink represents near target presentation inducing accommodation. Note the shift in the biopotentials when accommodation is triggered; (**b**) Example of a measurement run that was excluded from the training dataset, as no strong relation between the signal and accommodative demand can be seen; (**c**) Average values for biopotentials separated for different accommodation demands and training/excluded datasets. The difference in mean biopotential amplitude between disaccommodated state (0.2 D) and accommodated state (2.0 to 4.0 D) was higher for the training dataset. Error bars indicate 95% confidence intervals.

The time series biopotential signals from the previous study [31] were characterized using one far target accommodative demand at 0.2 D (5 m) and four near target accommodative demands at 2.0 D, 2.5 D, 3.0 D and 4.0 D (50 cm, 40 cm, 33 cm and 50 cm, respectively). A decline in signal amplitude was observed when accommodation was triggered by shifting the visual stimulus from the far target (0.2 D) to a near target (2–4 D) [31]. Manual inspection was used to identify samples suitable for training. Only those runs that exhibited notable changes in signal amplitude in response to accommodative demand change as in Figure 1a were selected for training the classifier, leading to the inclusion of 57 out of 145 measurement runs as training data. The measurement runs similar to Figure 1b were excluded, where the impact of change in accommodative demand is not visually evident in signal profile. Exclusion was likely necessitated by factors such as improper lens fit, lens decentralization following eye movements or blinks, and insufficient subject participation during certain runs. Figure 1c illustrates the differences in average signal levels for different accommodative demands. For the training data, there is larger difference between near (2.0 to 4.0 D) and far (0.2 D) target accommodative demands as compared to the excluded data.

After the selection of the training dataset, each sample was manually annotated into two classes to train the classifier: “accommodative change” and “accommodative no change”.

### 2.3. Preprocessing

**Figure 2:**
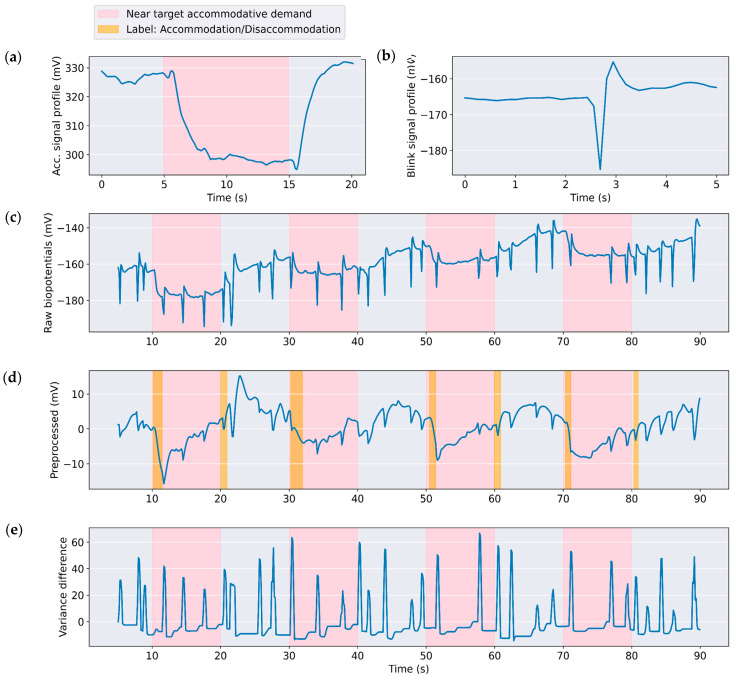
Typical profiles for: (**a**) Accommodation response for ciliary muscle raw biopotentials after change in demand. Note the Boxcar-shape pattern presented by the biopotentials, similar to the refraction profile following change in accommodation demand as shown by Shirachi et al. [36], but inverted in amplitude due to the electrode configuration; (**b**) Blink response for CM raw biopotentials characterized by a V-shaped pattern; (**c**) Example of raw biopotentials with frequent blink artifacts for a measurement run; (**d**) Preprocessed signal reduces the blink content and makes accommodative signals more apparent. The orange shade denotes annotated data as refractive change; (**e**) Augmented feature variance difference highlights the blink responses so enabling the classifier to learn non-accommodation-related events.

The accommodation response typically begins about 400 ms after a change in demand, with lens power adjustment starting within 500 ms and the entire process ideally completing in less than one second [36]. A typical accommodation demand-response curve is described by Shirachi et al. [36], where a boxcar-like pattern is observed during disaccommodation–accommodation–disaccommodation cycles. Correspondingly, the biopotential signals (Figure 2a) closely follow the accommodative response, while blinks (Figure 2b) appear different, exhibiting a V-shaped pattern. The preprocessing steps were designed to attenuate blinks and other artifacts, while preserving accommodation-related characteristics in the signal and using resource-efficient steps to reduce latency.

Schumayer et al. [31] identified the relevant range to be in the low-frequency range, between 0.025 and 0.575 Hz. A Butterworth (BW) second-order bandpass filter was used for preprocessing in that work, which was able to remove most of offset, baseline drift and higher frequency artifacts like blinks and power line interference.

It is important that data preparation before neural network training closely reflects the handling of real-time data during inference, when predictions are generated. Since, in real-time applications, the entire time series is not available at once, preprocessing was also performed on sequential data fragments rather than on the full time series. The BLE frame was reduced to 32 samples (corresponding to 128 ms at a sampling rate of 250 SPS). Using smaller frames resulted in insufficient processing time before subsequent data arrived, while larger frames increased end-to-end latency.

For the low-frequency range, a sample size of 32 (128 ms) was not sufficient for effective filtering. Therefore, a sequence of 256 such frames (32.768 s) was used for preprocessing and visualization of the biopotentials in the GUI during application. To mitigate filter edge effects, the data was padded by reflecting an additional eight seconds from the end, ensuring that the most recent frame did not lie at the sequence boundaries and was less susceptible to filtering artifacts. Zero-phase filtering was employed to maintain a linear phase response, further minimizing signal distortion.

Preprocessing involved a two-stage Butterworth filtering approach: firstly, a second-order bandpass filter with cutoff frequencies at 0.03 Hz and 0.8 Hz; secondly, a fifth-order lowpass filter at 0.4 Hz to attenuate rapid transients such as eye blinks. This cascade ensured strong attenuation above 0.4 Hz while preserving a flat passband response. The implementation utilized SciPy signal (The SciPy Community, Worldwide/Open Source) library in Python 3.10.5. Although finite impulse response (FIR) filters were considered, achieving comparable stopband attenuation required a high filter order (e.g., order ≥ 500 with a Hamming window), leading to increased computational load and latency. Ultimately, the chosen Butterworth filter cascade in SciPy provided an optimal balance between performance and efficiency for the preprocessing stage that was crucial for the overall pipeline.

### 2.4. Feature Engineering

Time series data differ from other types of data due the serially correlated, sequential and often multivariate nature of the data [37,38]. Due to the nature of our application, the classifier was required to provide continuous outputs by processing each incoming data frame based on short, recent windows, but still capture the time-related data dependencies. The univariate and sequential nature of the data, as well as the limited and constant size of each incoming data frame, necessitated strategies to enhance feature representation without significantly increasing data dimensionality.

Although variety of data augmentation and feature extraction methods exist, each suited to specific network architectures, two common approaches for deep learning discriminative models are: hand-engineered feature extraction, and the use of convolutional filters for automatic feature extraction [39,40]. A manual feature selection process was used to identify the 15 most relevant features, which were then used as inputs to a multilayer perceptron (MLP) classifier. The performance of various features was systematically evaluated by training models with different feature sets, and the final set was chosen to maximize model performance through benchmarking scores while ensuring that all features were computationally simple and efficient to extract.

The features are listed and briefly explained in Table 1. To capture both immediate and delayed temporal characteristics, features were extracted using two sample window sizes (32 and 96 samples), thereby accounting for recent signal changes and longer accommodative events observed in some subjects. The combination of two sample sizes and filter stages was used to generate a feature set comprising 15 features, each represented by a single scalar value.

Before the features were fed into the model, they were standardized to have a mean of zero and a standard deviation of one using a standard scaler. This normalization step ensured that all features contributed equally, preventing features with larger scales from dominating the learning process.

### 2.5. Deep Neural Network Classifier Architecture

Feature engineering enabled the use of a standard multilayer perceptron (MLP) classifier, which is a widely utilized deep neural network (DNN) architecture for classification tasks [41]. The network accepts 15 features as input and gives class probabilities for two output classes: accommodative change and no change.

**Figure 3:**
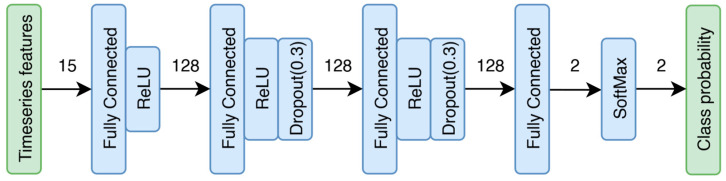
Architecture of the deep neural network. There are two fully connected (FC) hidden layers, input/out FC layers and a final SoftMax layer to convert logits to class probabilities. The size of the input and output dimensions for each stage is mentioned above the arrows.

The diagram (Figure 3) illustrates the complete model architecture. During training, the SoftMax layer is not used, whereas for inference, dropout layers are excluded. A rectified linear unit (ReLU) activation function was applied after the fully connected layers to introduce non-linearity, enabling the network to learn complex patterns within the data whereas dropout layers were also added to promote generalization and prevent overfitting [41]. PyTorch library (PyTorch Foundation, c/o the Linux Foundation, San Francisco, CA, USA) version 2.5.0 was used to train and deploy the model in Python. The model’s depth (number of hidden layers) and width (neurons per layer) were systematically optimized to ensure sufficient complexity for capturing essential data patterns, while avoiding overfitting and excessive memorization, thereby supporting robust predictions on unseen data. This optimization was achieved by training networks with varying architectures, depths, and widths, and assessing their performance on the validation dataset [41]. During the experiment in the retrospective study [31], participants were presented with a change in viewing target every ten seconds to consistently trigger accommodative changes. As accommodative responses typically last only about 500 ms [36], and changes were initiated every ten seconds, the resulting dataset was highly imbalanced between the change and no change classes. Hence, performance evaluation utilized confusion matrix-based metrics suitable for training with class imbalance: Precision, Recall, F1-score, Balanced Accuracy (BA), and Matthews Correlation Coefficient (MCC) [42,43,44].

The model was trained on 80 percent of the available data, with the remaining 20 percent reserved for validation [41]. Training was conducted over 200 epochs, with performance metrics for both test and validation datasets recorded every 50 epochs. This process was repeated for 20 iterations to check if model performance remained similar across different random initializations. A model was then selected for deployment based on the highest values in the scores for validation data. For benchmarking, the model’s performance was compared to several other classifiers, namely a deeper and wider MLP, a fully convolutional neural network (FCNN), a residual network (ResNet) as described by Wang et al. [37], and a hybrid architecture combining FCNN and MLP as described by Ignatov [40], and extreme gradient boosting (XGBoost) as described by Chen and Guestrin [45].

### 2.6. Decision Logic

The MLP classifier outputs a probability for each input data frame, corresponding to two possible classes. Since this is a binary classification task, only the probability associated with the change class is considered. This probability is then passed to a decision algorithm, which determines whether the most recent frame represents one of three final output classes: accommodation, disaccommodation, or no change.

To enhance the robustness and flexibility of the system for future implementation, a decision logic algorithm was added to generate the final classification output based on the current probability of change, first difference, and variance difference. Both first difference and variance difference were included among the input features during training, directly representing accommodative change and the presence of artifacts in the signal, respectively. The decision algorithm initially assigns the predicted class as no change. A classification of change is made only if both the probability of change and the first difference exceed their respective adjustable thresholds, while the variance difference remains below its threshold. Otherwise, the decision of no change is passed to the output. If a change is determined, the polarity of the first difference is used to distinguish between accommodation and disaccommodation. The thresholds and polarity of the first difference can be set during measurement.

### 2.7. Lens Tuning

The power of a tunable lens (EL16–40-TC-VIS-5D, Optotune AG, Dietikon, Switzerland) is changed depending to the output of the decision logic. If an accommodation or disaccommodation prediction is reached, the system checks if the current state of the tunable lens matches the predicted requirement. If there is a mismatch, the lens’s refractive power is adjusted.

The adjustment is performed using a programmable current source (ADN8810, Analog Devices, Wilmington, IL, USA), which controls the tunable lens. Instead of making a sudden change, the system increases or decreases the refractive power in small, discrete steps ranging from 0 D to 3 D. This transition occurs over a period of 500 milliseconds to 1 s, allowing for a gradual and more natural adjustment that mimics the eye’s physiological accommodation process. The exact duration of this adjustment can be tailored based on individual user comfort.

### 2.8. Full System Application and Simulation

**Figure 4:**
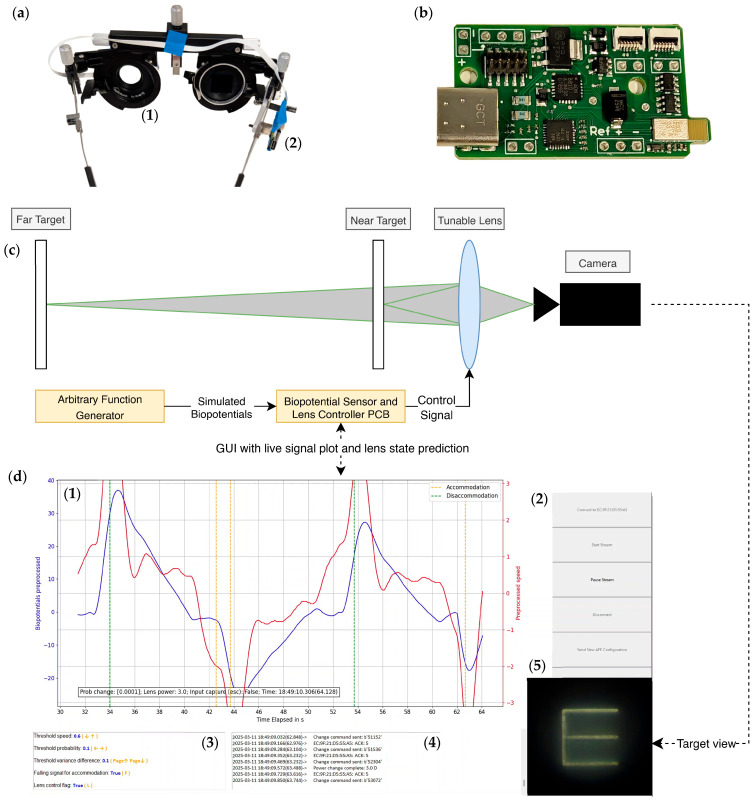
Application demonstration: (**a**) Trial frame with the tunable lens (**1**) for the left eye and the sensor and lens controller circuit boards (**2**) on the right side; (**b**) Biopotential sensor and lens controller PCB; (**c**) Optical bench simulation setup. Biopotentials are generated using an arbitrary function generator, which provides the same signal output as measured in the previous study. A camera captures the subject’s view, and both near and far targets are present. The tunable lens selectively focuses on one of the targets depending on the system’s prediction. (**d**) Control GUI displaying the live signal and prediction plot (**1**), control buttons (**2**) on the right, threshold parameters window (**3**) on the bottom left, log output window (**4**) at the bottom center, and view of the targets (**5**) at the bottom right, captured by the camera in the simulation setup. The blue curve represents biopotentials (mV), while the red curve shows their first difference (mV). A dotted vertical orange line indicates that accommodation is predicted by the system, whereas a dotted vertical green line indicates disaccommodation.

The complete system acquires biopotential signals from contact lens electrodes, which are processed by the sensor and lens controller PCB. This PCB (Figure 4b) serves both as the input interface for signal acquisition and as the output controller for lens power adjustments. Initially, the input signals are transmitted to a BLE central computer, where the application performs signal preprocessing and feeds the processed data to a neural network model. After the final decision logic generates a prediction, the system issues a command to the PCB to adjust the lens power if necessary. To access the functionality with presbyopic subjects, a wearable setup with the tunable lens and the biopotential sensor and lens controller PCB (Figure 4a) was integrated into a trial frame. The application GUI does the preprocessing, hosts the predictive model and decision logic and displays a live signal acquisition plot as shown in Figure 4d. The GUI also provides control buttons for operator input and an output log window to provide information of the system’s status.

The system was validated on an optical bench setup prior to testing with presbyopic subjects. Two illuminated targets positioned at 1 D and 3 D (100 cm and 33 cm, respectively), as shown in Figure 4c. An arbitrary function generator (33500b, Keysight Technologies, Santa Rosa, CA, USA) delivered pre-recorded biopotential signals to the PCB, which relayed them to the application. The application issued lens adjustment commands as required, allowing the tunable lens to bring either the near or far target into focus for the camera. The camera feed displayed at the bottom right (Figure 4d) was used exclusively during simulation, acting as the subject view.

A video illustrating the simulation, which includes both the control application GUI and the camera capture (Figure 4d) is available in the Supplementary Materials (Video S1). A reduction in amplitude of the biopotential signal triggered accommodation, bringing the optotype E facing right (near target) into focus, whereas an increase in amplitude resulted in the prediction of disaccommodation that focused on the optotype E facing upwards (far target). A stepwise change in the tunable lens power was observed, gradually bringing different optotypes at different distances into focus. Lens power changes were inferred every 10 s, consistent with the protocol used during the experiment for the previous study.

### 2.9. Preliminary Evaluation with Presbyopic Subjects

Two presbyopic volunteers participated in the preliminary evaluation [35], one male (aged 79 years) and one female (aged 76 years), each with best-corrected visual acuity (BCVA) of 20/25 or better. Visual acuity testing was performed using an optical setup adapted from Wagner et al. [46], and accommodative stimuli was presented at two distinct viewing distances: 500 cm (far target) and 33 cm (near target). The stimuli consisted of four randomly selected Sloan letters, which were displayed in a controlled environment using the PsychoPy (University of Nottingham, Nottingham, UK) software package (version 2022.2.4).

The participants were asked to identify the letters and for each viewing distance, the size of the presented letters was adaptively adjusted to determine the participant’s visual acuity threshold. This was achieved using an interleaved adaptive staircase algorithm (Quaternion Estimator; QUEST), with the procedure continuing until the estimated threshold’s confidence interval width reached 0.2 logMAR. Both participants completed two separate test runs, once where the lens was set at 0 D (lens off), and once with active correction between 0 D and 3 D using the tunable lens (lens on).

### 2.10. Use of Generative Artificial Intelligence (GenAI)

OpenAI ChatGPT v3.5 and GitHub Copilot powered by OpenAI GPT-4.1 were utilized in this work. Both tools were employed for developing the code for training and deployment with a GUI, while Copilot was also used for data analysis and text refinement for this paper.

## 3. Results

### 3.1. Performance of the Proposed MLP Model

**Figure 5:**
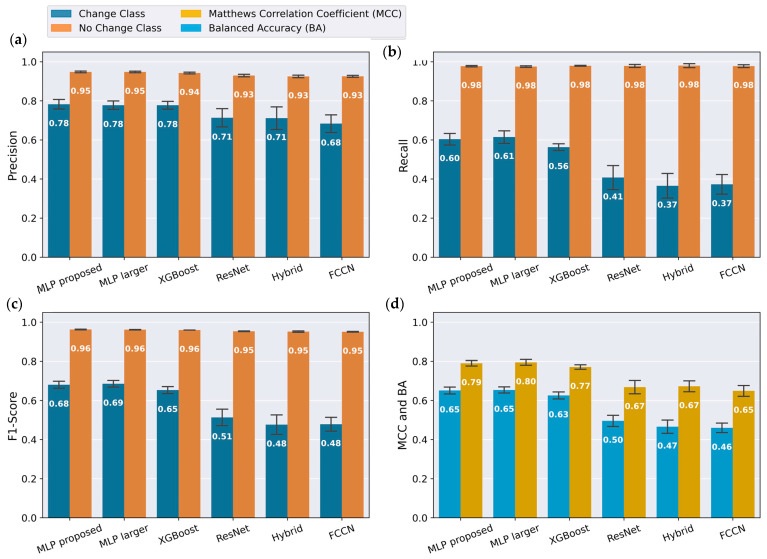
Performance evaluation of different neural network architectures. (**a**) Precision; (**b**) Recall; (**c**) F1-score; (**d**) Balanced Accuracy (BA) and Matthews Correlation Coefficient (MCC) for the proposed model and other comparison models. The larger MLP, FCCN, and ResNet architectures are described by Wang et al. [37], the hybrid model is described by Ignatov [40], while the XGBoost model is described by Chen and Guestrin [45]. Bars represent mean values, while error bars indicate standard deviations computed over 20 independent random initializations.

The proposed MLP model was compared with the three models mentioned by Wang et al. [37], and one hybrid model mentioned by Ignatov [40]. Figure 5 shows the precision, recall, F1-score, Balanced Accuracy (BA) and Matthews Correlation Coefficient (MCC) of all the compared models with the same training data. It achieved an F1-score of 0.96 for the change class, 0.78 for the no change class, BA of 0.79 and MCC of 0.65 across both classes. For the no change class, all models exhibited similar performance, with F1-scores around 0.95. The models with convolutional layers had a decrease in the F1-score for the change class, which dropped to approximately 0.5. The XGBoost and wider and deeper MLP architecture showed similar performance as the proposed model across all evaluation parameters. On a notebook computer (Katana GF66, Micro Star International, New Taipei City, Taiwan) equipped with a real-time plotting GUI, the average processing time per frame was 13 ms, with a maximum of 190 ms recorded during a 5 min session.

### 3.2. Performance in Terms of Tunable Lens State

**Figure 6:**
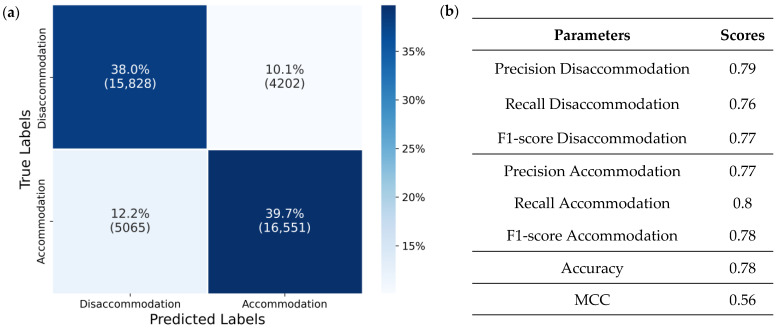
Performance metrics for output classes: Accommodation and disaccommodation for the tunable lens state. (**a**) Confusion matrix; (**b**) Evaluation parameters derived from the confusion matrix.

The final system output is the state of the tunable lens. Figure 6a presents a confusion matrix, illustrating the number of accommodation and disaccommodation events that were correctly classified across the entire original dataset. Of 21,616 accommodation events (51.9% of all data), 16,551 (76% of accommodation) and out of 20,030 (48.1% of all data) disaccommodation samples, 15,828 (79% of disaccommodation) were correctly classified. The performance metrics derived from the confusion matrix for the tunable lens state are shown in Figure 6b. In the case of the tunable lens state, the two classes are nearly balanced, and comparable performance values are observed for each class. The final accuracy is 0.78, with an MCC score of 0.55.

### 3.3. Visual Acuity Measurements for Two Presbyopic Subjects

**Figure 7:**
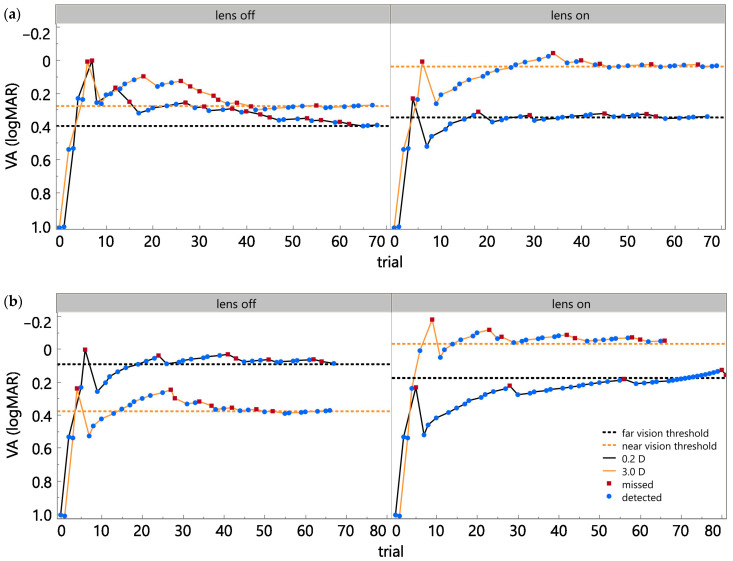
Visual acuity measurements for (**a**) Subject 1; and (**b**) Subject 2 under fixed (lens off) and active (lens on) lens settings [35]. For both subjects, measured near vision improved with the tunable lens activated, whereas distance visual acuity thresholds remained comparable across lens conditions.

Visual acuity thresholds for two subjects are presented in Figure 7, with measurements at near (orange) and distance (black) viewing conditions [35]. The horizontal dotted orange lines denote near visual acuity thresholds with the tunable lens activated (lens on) at the right side, while the fixed lens condition (lens off) is indicated at the left side for comparison. For both subjects, near visual acuity was better when the tunable lens was activated, as shown by the upward shift in the orange line at near viewing distance. In contrast, distance visual acuity thresholds remained similar across lens conditions. Specifically, Subject 1 demonstrated an improvement of 0.24 logMAR while Subject 2 exhibited an improvement of 0.41 logMAR at near distance with the tunable lens activated [35].

## 4. Discussion

In this work, a neural network model for the control of an artificial lens based on neuromuscular signals recorded from the ciliary muscle during accommodation was developed, with the aim to restore dynamic accommodation in presbyopia. Different models were trained and compared using data from a retrospective study.

### 4.1. Performance Analysis

Deep learning methods for TSC have gained substantial traction in recent years, enabled by open-source libraries, advances in feature extraction and transformation, and architectural innovations so that they are now applied across a wide range of real-world problems [37,39,40,41,47]. The models evaluated in this study exhibited varying accuracies across output classes, which can be primarily attributed to class imbalance and a higher prevalence of artifacts in the minority class. This trend is evident in Figure 5a–c, where the orange bars representing the change class are smaller than the blue bars representing the no change class. For the final output state of the tunable lens, accommodation or disaccommodation, the performance scores are comparable, as seen in Figure 6b. This can be explained by the balanced quantity of data for these states, and that the artifacts similarly affect both transitions between change and no change.

Figure 5c illustrates that the models with convolutional layers (FCCN, ResNet, and Hybrid) resulted in decreased output accuracy, particularly for the class change. Although network architectures with convolutional layers are widely used due to their ability to perform automatic feature extraction, they were less suitable in this context. In this implementation, targeted preprocessing and the selection of custom features yielded better results than automated feature learning. Furthermore, machine learning algorithms generally achieve optimal performance when their capacity matches the complexity of the problem and the available training data [41]. When models exhibit similar performance, it is preferable to choose the simpler one, as it is less likely to overfit and more likely to generalize well to unseen data [41]. Since the proposed MLP performed as well as the deeper and wider MLP and XGBoost while offering better scalability, it was selected.

A sample size of 32 with 250 SPS meant that there was an incoming data frame every 128 ms. It is important to keep the sample size low to keep the output latency low. Even though the processing latency for a frame in the deployment software is 13 ms on average, the delivery of the data to the application layer from the physical layer may be higher due to the operating system stack and system scheduling. Empirical testing demonstrated that using smaller sample sizes resulted in increased system lag over time, with predictions becoming progressively delayed and failing to be generated in real time.

### 4.2. Application Considerations and Possible Impacts

Accommodation and disaccommodation involve the contraction and relaxation of the ciliary muscle, respectively. In our approach, the biopotentials emitted by the ciliary muscle are used as signals to predict the refractive state of the eye. These biopotentials are regulated by neural mechanisms of the visual system, which coordinate the rapid adjustments necessary to maintain clear vision at varying distances. The process of accommodation functions as a feedback system [11,24,48], where the eye continuously compares the desired focal state with the actual focus and adjusts the lens accordingly, a process reflected in the ciliary muscle’s neuromuscular signals. It is also argued that micro fluctuations in accommodation with a low-frequency component (less than 0.6 Hz) are crucial in maintaining a steady-state accommodation, but these fluctuations are found to be suppressed in old-age [49,50]. A balanced accuracy of 0.79 for the deployed model in an application such as smart adaptive spectacles may not appear ideal at first glance. However, it is important to note that this value reflects offline prediction accuracy. In the real-time system, with new frames arriving every 192 ms, missed or misclassified frames are likely to be quickly corrected by subsequent predictions. Instantaneous misclassifications, which often result from blink or eye movement artifacts, should be followed by accurate predictions once the artifact subsides, allowing the system to promptly revert the lens to its intended state. The slow, stepwise transition in lens power further ensures that any unintended changes are brief and incremental. This approach may work better with the active, closed-loop nature of the visual system, potentially mimicking accommodative microfluctuations that are reduced in presbyopic individuals. This helps the visual system work together with the lens, rather than against it as micro-movements in focal power align with the natural behavior of the visual system. Moreover, gradual incremental changes can facilitate user adaptation by making it easier to perceive and understand the connection between their own control signals and lens adjustments, potentially improving comfort and reducing the learning curve. As a result, the effective system accuracy experienced by the user is likely to be higher in practice, although it might highly depend and vary individually on the dynamic lens tuning characteristics as well.

Although this work involves a passive contact lens sensor, smart contact lenses (SCLs) have been extensively investigated as both sensors and fully integrated wireless platforms for non-invasive, continuous eye and systemic health monitoring. These devices incorporate micro- and nanoscale sensors within biocompatible materials, enabling real-time detection of biomarkers such as intraocular pressure and tear glucose levels [51]. Despite significant remaining challenges, including biocompatibility, power supply, device miniaturization, detection sensitivity, long-term stability, and data privacy, SCLs present substantial potential, particularly when combined with machine learning and the Internet of Things (IoT) [52]. Liu et al. [53] provide a comprehensive overview of strategies for deploying SCLs in multifunctional medical applications, emphasizing recent advancements and suggesting several future directions that may inform further development and of the proposed accommodating lens system.

### 4.3. Limitations and Possible Improvements

A primary limitation of this approach is that accurate control is dependent on signal quality, which is influenced by subject participation and the fit of the lens. Utilizing hard scleral contact lenses introduces an inherent risk of mismatch between the corneal profile and the lens, and lens decentralization following eye movements and blinks, potentially affecting system accuracy. Eye squinting was observed to be prolonged and have variable signal characteristics, which may introduce frequency components similar to those associated with accommodation and negatively impact the prediction accuracy. However, it is important to recognize that accommodation itself is a dynamic and self-correcting process, guided by continuous retinal blur detection and subsequent adjustment of ciliary muscle activity [11,24]. Consequently, occasional misclassifications resulting in transient accommodation errors are expected to be rectified through ongoing feedback mechanisms, akin to the natural accommodative process. For signal reception directly from the ciliary muscle and lower impact of artifacts and lens fit, an implantable system using intraocular ring electrode [54] is currently under development for study in nonhuman primates.

One of the key methodological challenges in this work is that signal acquisition was initially performed in emmetropic subjects to characterize the biopotentials of the ciliary muscle, and this dataset was subsequently used to train a model intended for application in presbyopes. Previous work [55] addressed this topic through a study involving presbyopic subjects, providing a valuable foundation for further research. However, subsequent updates to the measurement setup and lens design, implemented to enhance data quality, prevented the use of those earlier data for the current model training. The current decision logic enhances flexibility by integrating the ANN model output with additional criteria, the first difference (as a measure of signal speed) and variance difference (for blink artifact detection). This approach ensures that predictions are not solely dependent on the original training dataset derived from emmetropic subjects. Future research will focus on characterizing biopotentials directly in presbyopic subjects, thereby providing a more relevant dataset for continued model development.

Measurement runs exhibiting a very low signal-to-noise ratio, manifested by signal amplitude changes that did not correspond to changes in accommodative demand, were excluded through visual inspection. Although a more systematic and objective approach using statistical parameters for run classification is preferable and planned for future work, manual selection was adopted here due to the limited dataset size, particularly for the minority class.

The system’s behavior under active lens control conditions remains unknown. During the collection of emmetrope data, a tunable lens was not used, and in simulation experiments, the neural mechanisms within the visual system were absent. Introducing adaptive lens adjustment with the predictive system while capturing ciliary muscle biopotentials simultaneously may alter key signal characteristics. Consequently, the visual system’s natural accommodation response may conflict with artificial lens adjustments, leading to potential challenges such as over-correction or time mismatches between the responses. Such conditions may introduce positive feedback, resulting in instability and undesired oscillations in lens power. Strategies such as decreasing prediction latency, implementing accommodation lag in the external lens, and leveraging user adaptation or training effects may help mitigate these risks of instability. Systematic evaluation of visual performance and user experience under real-world, dynamic conditions, including metrics such as comfort, glare, and lens handling, is critical for future development and possible clinical translation of the technology.

Further availability of data and testing in presbyopic subjects will improve insights into the overall performance and behavior within a closed-loop accommodative system. Currently, the system has been tested in two presbyopic subjects, with preliminary results demonstrating substantial improvements in near-target visual acuity (0.24 logMAR and 0.41 logMAR) [35]. No considerable change was observed in far-target visual acuity, which is consistent with the system’s design to selectively enhance near vision in presbyopes [35]. Such improvements in near vision suggest that the feedback loop for visual accommodation can be re-established using the proposed system. Nevertheless, further experiments with more participants are needed to confirm these findings and assess the generalizability of the proposed system.

The present work utilizes a benchtop prototype designed for short-term use in experimental settings, wherein the sensing, processing, and actuation modules are implemented separately. This configuration was intended for focused validation of the control algorithms using the recording system and data collected in a prior study that incorporated a commercially available tunable lens. Achieving full system integration within a wearable contact lens remains a substantial engineering challenge, primarily due to current limitations in power supply, device miniaturization, and on-lens processing capabilities. With recent advances in lens design, energy sources, and ANN deployment technologies, ongoing research is directed toward achieving an integrated and miniaturized contact or implantable lens system. Although these results indicate that changes in lens fit parameters like vault height and centration can impact system performance, custom fitting, potentially guided by ocular topography, may be essential for future applications. Additionally, subsequent research could investigate flexible lens designs to reduce the reliance on exact lens positioning.

## 5. Conclusions

Presbyopia is an age-related condition characterized by the gradual loss of the eye’s ability to accommodate, resulting in impaired near vision. This common disorder significantly affects daily activities and quality of life, as tasks requiring clear near vision become increasingly difficult. Common correction methods, such as reading glasses, bifocals, and multifocal contact lenses, often present disadvantages including limited visual flexibility, dependency on external devices, and limited adaptation to dynamic accommodative demands.

The present work introduces a novel approach for presbyopic correction using a closed-loop system that utilizes a deep neural network model and inherent visual pathways to predict the eye’s accommodative state in real time. By utilizing ciliary muscle biopotentials, the proposed system has the potential for adaptive control of an external lens, with the aim of restoring near vision. The system demonstrated a balanced accuracy of 0.79 and clear improvements in near-target visual acuity (0.24 and 0.41 logMAR) in initial tests with two long-term presbyopic subjects.

This proof-of-concept shows considerable promise for transforming presbyopia management. Unlike traditional corrections, the proposed model offers quasi-natural, dynamic adaptation of lens power, potentially allowing users to regain a degree of control over their visual focus through neuroadaptive processes. The slow, incremental lens transitions further improve comfort and robustness, minimizing abrupt changes, stimulating microfluctuations and reducing the impact of occasional misclassifications.

Future work will focus on expanding measurements in presbyopic subjects, refining model architectures, and optimizing signal processing techniques to further enhance real-time accuracy and user comfort. Continued development and evaluation of the proposed system may enable restoration of near vision that closely resembles youthful accommodation in individuals with presbyopia, ultimately contributing to improved visual function and quality of life for older adults.

## Figures and Tables

**Table 1:** List of extracted features, categorized according to filter output stages and sample size.

Filter Stage	Sample Size	Feature	Description
First	32	filtered_speed_32	Average first difference for 32 samples
		filtered_acceleration_32	Average second difference for 32 samples
		filtered_variance_32	Average variance for 32 samples
	96	filtered_speed_96	Average first difference for 96 samples
		filtered_acceleration_96	Average second difference for 96 samples
		filtered_variance_96	Average variance for 96 samples
Second	32	preprocessed_biopotentials_32	Average signal value for 32 recent samples
		preprocessed_speed_32	Average first difference for 32 samples
		preprocessed_acceleration_32	Average second difference for 32 samples
		preprocessed_variance_32	Average variance for 32 samples
		variance difference_32	Difference in variance for two filter stages output
	96	preprocessed_speed_96	Average first difference for 96 samples
		preprocessed_acceleration_96	Average second difference for 96 samples
		preprocessed_variance_96	Average variance for 96 samples
		variance_difference_96	Difference in variance for two filter stages output

## Data Availability

The raw data supporting the conclusions of this article will be made available by the authors on request.

## Supplementary Materials

The following supporting information can be downloaded at: https://www.mdpi.com/article/10.3390/bioengineering12111228/s1, Video S1: Simulation with Control GUI and Wearer View.

## Abbreviations

The following abbreviations are used in this manuscript:

FDA	Food and Drug Administration
LiDAR	Light Detection and Ranging
MTLCL	Matrices of Tunable Liquid-Crystal Lenses
IOL	Intraocular Lens
SPS	Samples Per Second
AFE	Analog Front-End
BLE	Bluetooth Low Energy
BW	Butterworth
GUI	Graphical User Interface
PCB	Printed Circuit Board
MLP	Multilayer Perceptron
DNN	Deep Neural Network
TSC	Timeseries Classification
FC	Fully Connected
ReLU	Rectified Linear Unit
BCVA	Best-Corrected Visual Acuity
QUEST	Quaternion Estimator
BA	Balanced Accuracy
MCC	Matthews Correlation Coefficient
FCNN	Fully Convolutional Neural Network
ResNet	Residual Network
XGBoost	eXtreme Gradient Boosting
GenAI	Generative Artificial Intelligence (GenAI)
SCL	Smart Contact Lens

## References

[1] Charman W.N. (2008). The Eye in Focus: Accommodation and Presbyopia. Clin. Exp. Optom..

[2] Heys K.R., Cram S.L., Truscott R.J.W. (2004). Massive Increase in the Stiffness of the Human Lens Nucleus with Age: The Basis for Presbyopia?. Mol. Vis..

[3] Fisher R.F. (1969). Elastic Constants of the Human Lens Capsule. J. Physiol..

[4] Fricke T.R., Tahhan N., Resnikoff S., Papas E., Burnett A., Ho S.M., Naduvilath T., Naidoo K.S. (2018). Global Prevalence of Presbyopia and Vision Impairment from Uncorrected Presbyopia. Ophthalmology.

[5] Duane A. (1912). Normal Values of Accommodation at All Ages. JAMA.

[6] Helmholtz H. (1855). Ueber die Accommodation des Auges. Graefes Arch. Clin. Exp. Ophthalmol..

[7] Von Helmholtz H., Southall J.P.C. (1924). Mechanism of Accommodation. Helmholtz’s Treatise on Physiological Optics (Trans. from the 3rd German ed.).

[8] Atchison D.A. (1995). Accommodation and Presbyopia. Ophthalmic Physiol. Opt..

[9] Goldberg D.B. (2015). Computer-Animated Model of Accommodation and Presbyopia. J. Cataract Refract. Surg..

[10] Zuo H., Cheng H., Lin M., Gao X., Xiang Y., Zhang T., Gao N., Du M., Chen Y., Zheng S. (2024). The Effect of Aging on the Ciliary Muscle and Its Potential Relationship with Presbyopia: A Literature Review. PeerJ.

[11] Bharadwaj S.R. (2025). Ocular Accommodation: The Autofocus Mechanism of the Human Eye. Annu. Rev. Vis. Sci..

[12] Fisher R.F. (1988). The Mechanics of Accommodation in Relation to Presbyopia. Eye.

[13] Strenk S.A., Strenk L.M., Koretz J.F. (2005). The Mechanism of Presbyopia. Prog. Retin. Eye Res..

[14] Wolffsohn J.S., Davies L.N. (2019). Presbyopia: Effectiveness of Correction Strategies. Prog. Retin. Eye Res..

[15] Tabernero J., Chirre E., Hervella L., Prieto P., Artal P. (2016). The Accommodative Ciliary Muscle Function Is Preserved in Older Humans. Sci. Rep..

[16] Charman W.N. (2014). Developments in the Correction of Presbyopia I: Spectacle and Contact Lenses. Ophthalmic Physiol. Opt..

[17] Charman W.N. (2014). Developments in the Correction of Presbyopia II: Surgical Approaches. Ophthalmic Physiol. Opt..

[18] Wolffsohn J.S., Davies L.N., Sheppard A.L. (2023). New Insights in Presbyopia: Impact of Correction Strategies. BMJ Open Ophth..

[19] Zhang X., Xiong X., Zhang H., Huang T., Zhou X. (2024). Pilocarpine in the Treatment of Presbyopia: Progress, Issues, and Future Prospects. Drugs Aging.

[20] Karkhanis M.U., Ghosh C., Banerjee A., Hasan N., Likhite R., Ghosh T., Kim H., Mastrangelo C.H. (2022). Correcting Presbyopia With Autofocusing Liquid-Lens Eyeglasses. IEEE Trans. Biomed. Eng..

[21] Agarwala R., Severitt B.R., Reichel F.F., Hosp B.W., Wahl S. (2025). Performance of Focus-Tunable Presbyopia Correction Lenses Operated Using Gaze-Tracking and LIDAR. Biomed. Opt. Express.

[22] Bégel L., Galstian T. (2025). Dynamic Presbyopia Correction in the Macular Field of View by Using a Liquid Crystal Lens. Biomed. Opt. Express.

[23] Fincham E.F., Walton J. (1957). The Reciprocal Actions of Accommodation and Convergence. J. Physiol..

[24] Toates F.M. (1970). A Model of Accommodation. Vis. Res..

[25] Toates F.M. (1972). Accommodation Function of the Human Eye. Physiol. Rev..

[26] Dick H.B., Gerste R.D. (2021). Future Intraocular Lens Technologies. Ophthalmology.

[27] Schubert G. (1955). Aktionspotentiale des M. ciliaris beim Menschen. Graefes. Arch. Clin. Exp. Ophthalmol..

[28] Alpern M., Ellen P., Goldsmith R.I. (1958). The Electrical Response of the Human Eye in Far-to-Near Accommodation. Arch. Ophthalmol..

[29] Jacobson J.H., Romaine H.H., Halberg G.P., Stephens G. (1958). The Electric Activity of the Eye During Accommodation. Am. J. Ophthalmol..

[30] Hagiwara H., Ishikawa S. (1962). The Action Potential of the Ciliary Muscle. Ophthalmologica.

[31] Schumayer S., Sigdel B., Jarboui M.A., Zrenner E., Bucher V., Straßer T., Wagner S. (2025). Non-Invasive Measuring of Biopotentials of the Ciliary Muscle during Accommodation in Emmetropes. Sci. Rep..

[32] Schumayer S., Simon N., Sittkus B., Wagner S., Bucher V., Strasser T. (2022). Novel Three-Dimensional and Biocompatible Lift-Off Method for Selective Metallization of a Scleral Contact Lens Electrode for Biopotential Detection. Front. Med. Technol..

[33] Wagner S., Zrenner E., Strasser T. (2018). Ciliary Muscle Thickness Profiles Derived from Optical Coherence Tomography Images. Biomed. Opt. Express.

[34] Kaltenstadler S., Sigdel B., Schumayer S., Steinhoff R., Straßer T., Rothermel A. An Implantable Ciliary Muscle LFP Recording and Transmitting System. Proceedings of the 2024 46th Annual International Conference of the IEEE Engineering in Medicine and Biology Society (EMBC).

[35] Schumayer S., Sigdel B., Nikolaidou A., Wolfram L., Wagner S., Zrenner E., Bucher V., Straßer T. Preserved Ciliary Muscle Biopotentials Enable Artificial Lens Control and near Vision Recovery in Long-Term Presbyopia. Proceedings of the 62nd Annual Symposium of the International Society for Clinical Electrophysiology of Vision (ISCEV 2025).

[36] Shirachi D., Liu J., Lee M., Jang J., Wong J., Stark L. (1978). Accommodation Dynamics I. Range Nonlinearity. Am. J. Optom. Physiol. Opt..

[37] Wang Z., Yan W., Oates T. Time Series Classification from Scratch with Deep Neural Networks: A Strong Baseline. Proceedings of the 2017 International Joint Conference on Neural Networks (IJCNN).

[38] Sarker I.H. (2021). Deep Learning: A Comprehensive Overview on Techniques, Taxonomy, Applications and Research Directions. SN Comput. Sci..

[39] Ismail Fawaz H., Forestier G., Weber J., Idoumghar L., Muller P.-A. (2019). Deep Learning for Time Series Classification: A Review. Data Min. Knowl. Discov..

[40] Ignatov A. (2018). Real-Time Human Activity Recognition from Accelerometer Data Using Convolutional Neural Networks. Appl. Soft Comput..

[41] Goodfellow I., Courville A., Bengio Y. (2016). Deep Learning.

[42] Hammad M., Alkinani M.H., Gupta B.B., Abd El-Latif A.A. (2022). Myocardial Infarction Detection Based on Deep Neural Network on Imbalanced Data. Multimed. Syst..

[43] Bekkar M., Djemaa D.H.K. (2013). Evaluation Measures for Models Assessment over Imbalanced Data Sets. J. Inf. Eng. App..

[44] Jeni L.A., Cohn J.F., De La Torre F. (2013). Facing Imbalanced Data--Recommendations for the Use of Performance Metrics. Proceedings of the 2013 Humaine Association Conference on Affective Computing and Intelligent Interaction.

[45] Chen T., Guestrin C. (2016). XGBoost: A Scalable Tree Boosting System. Proceedings of the Proceedings of the 22nd ACM SIGKDD International Conference on Knowledge Discovery and Data Mining.

[46] Wagner S., Schaeffel F., Zrenner E., Straßer T. (2019). Prolonged Nearwork Affects the Ciliary Muscle Morphology. Exp. Eye. Res..

[47] Faouzi J. (2024). Time Series Classification: A Review of Algorithms and Implementations. Time Series Analysis—Recent Advances, New Perspectives and Applications.

[48] Read J.C.A., Kaspiris-Rousellis C., Wood T.S., Wu B., Vlaskamp B.N.S., Schor C.M. (2022). Seeing the Future: Predictive Control in Neural Models of Ocular Accommodation. J. Vis..

[49] Charman W.N., Heron G. (2015). Microfluctuations in Accommodation: An Update on Their Characteristics and Possible Role. Ophthalmic Physiol. Opt..

[50] Rohman L., Ruggeri M., Ho A., Parel J.-M., Manns F. (2023). Lens Thickness Microfluctuations in Young and Prepresbyopic Adults During Steady-State Accommodation. Investig. Ophthalmol. Vis. Sci..

[51] Kazanskiy N.L., Khonina S.N., Butt M.A. (2023). Smart Contact Lenses—A Step towards Non-Invasive Continuous Eye Health Monitoring. Biosensors.

[52] Han F., Ge P., Wang F., Yang Y., Chen S., Kang J., Ren Y., Liu H., Wei Z., He Y. (2024). Smart Contact Lenses: From Rational Design Strategies to Wearable Health Monitoring. Chem. Eng. J..

[53] Liu X., Ye Y., Ge Y., Qu J., Liedberg B., Zhang Q., Wang Y. (2024). Smart Contact Lenses for Healthcare Monitoring and Therapy. ACS Nano.

[54] Schumayer S., Zahrani E.G., Azarhoushang B., Bucher V., Straßer T. (2025). Design and In Vivo Evaluation of an Intraocular Electrode for Ciliary Muscle Biopotential Measurement in a Non-Human Primate Model of Human Accommodation. Biosensors.

[55] Clouse M.M. (2017). Recording and Processing of Tissue-Specific Ocular Electrical Biosignals for Applications in Biomedical Devices. Ph.D. Thesis.

